# Iatrogenic left main-stem dissection extending to the circumflex artery and retrogradely involving the left and non-coronary sinuses of Valsalva: iatrogenic aortocoronary dissection

**DOI:** 10.5830/CVJA-2015-060

**Published:** 2015

**Authors:** Radosław Zwoliński, Anna Marcinkiewicz, Ryszard Jaszewski, Konrad Szymczyk, Robert Pietruszyński

**Affiliations:** Department of Cardiac Surgery, Clinical Teaching Centre, Medical University of Lodz, Lodz, Poland; Department of Cardiac Surgery, Clinical Teaching Centre, Medical University of Lodz, Lodz, Poland; Department of Cardiac Surgery, Clinical Teaching Centre, Medical University of Lodz, Lodz, Poland; Department of Radiology: Imaging Diagnostics, Norbert Barlicki Memorial Teaching Hospital No 1, Medical University of Lodz, Lodz, Poland; Department of Vascular Diagnostics and Procedures, Military Teaching Hospital, Veterans Central Hospital, Lodz, Poland

**Keywords:** left main-stem dissection, limited aortic dissection, coronary angiography

## Abstract

We present the case of a 57-year-old female who experienced iatrogenic left main-stem (LMS) dissection during elective coronary angiography. The dissection immediately affected the circumflex artery (Cx), causing its total distal occlusion, and the left anterior descending artery (LAD), in which a metal stent, implanted six months earlier, provided blood flow. The dissection spread retrogradely to the left and non-coronary sinuses of Valsalva (SV). Ventricular fibrillation (VF) occurred but the patient was successfully defibrillated. The subsequent introduction of a catheter resulted in recurrent VF, again successfully defibrillated. Total arterial myocardial revascularisation with double skeletonised internal thoracic arteries was performed without complications and SV repair was avoided. At the one-year follow up, a control multi-slice CT (MSCT) angiography was conducted, revealing complete healing of the SV and LMS dissections. It also showed native blood flow, the left internal thoracic artery (LITA) graft to the Cx occlusion, and a patent right internal thoracic artery (RITA) graft implanted to the LAD.

## Abstract

According to the simplified classification, iatrogenic left main-stem (LMS) dissection involving the aortic root is the rarest and most life-threatening type of dissection. The incidence of iatrogenic aortic root dissection is estimated to be 0.02%. In the majority of cases it remains confined to the coronary sinus of Valsalva (SV).^1^

Although therapeutic management of LMS dissection involving SV is demanding, there are few published works in this field.^2^-^4^ There are neither guidelines nor consensus among experts on how to manage LMS and retrograde SV dissection during percutaneous procedures. The published literature on iatrogenic dissection describes the following therapeutic solutions: surgical revascularisation with SV repair, including usage of an autologous patch; percutaneous angioplasty with stent(s) implantation to cover the ostial dissection; and conservative treatment, which can provide spontaneous healing of the dissection.^1^-^4^

## Case report

A 57-year-old patient with well-controlled arterial hypertension, hyperlipidaemia and anterolateral ST-elevation myocardial infarction (STEMI) was treated with a primary bare-metal stent (BMS) implantation to the left anterior descending artery (LAD). Six months later, she underwent an elective coronary angiography due to exacerbated angina pectoris. On admission, she was classified as class III according to the Canadian Cardiovascular Society classification (CCS).

**Fig. 1.**
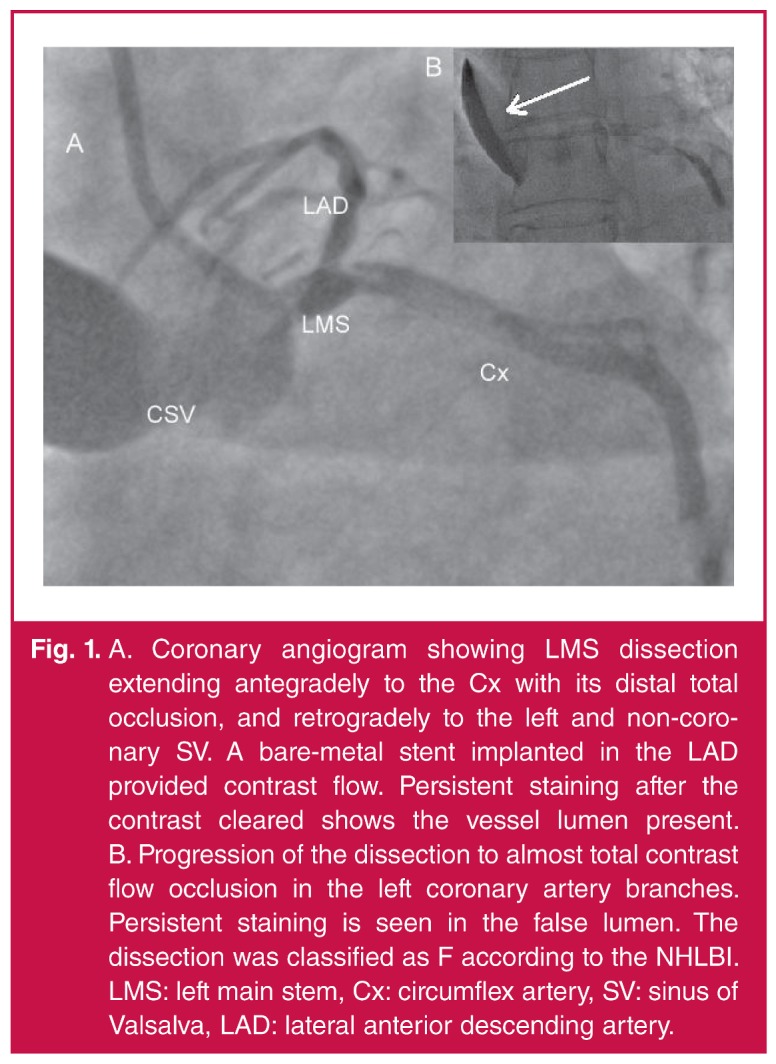
A. Coronary angiogram showing LMS dissection extending antegradely to the Cx with its distal total occlusion, and retrogradely to the left and non-coronary SV. A bare-metal stent implanted in the LAD provided contrast flow. Persistent staining after the contrast cleared shows the vessel lumen present. B. Progression of the dissection to almost total contrast flow occlusion in the left coronary artery branches. Persistent staining is seen in the false lumen. The dissection was classified as F according to the NHLBI. LMS: left main stem, Cx: circumflex artery, SV: sinus of Valsalva, LAD: lateral anterior descending artery.

During coronary angiography, the angiogram showed both the positive long-term effect of the BMS implantation and the absence of any significant progression of previously described atherosclerotic lesions. After completing the left coronary artery examination, the patient started complaining of chest pain. At the same time the electrocardiogram (ECG) showed ST-segment elevation. As the patient became symptomatic, catheterisation of the left coronary artery was repeated. A standard diagnostic coronary catheter, Impuls 6-Fr JL3.5 Boston (guidewire 6-Fr JL3.5 Medtronic Launcher) was used. This time the examination revealed LMS dissection, antegrade dissection of the circumflex artery (Cx) causing distal occlusion of blood flow, and contrasting of the left aortic bulb (Fig. 1). Instant ventricular fibrillation (VF) occurred but the patient was successfully defibrillated. The cardiologist immediately attempted to cover the dissection with a stent, but VF recurred, and was again successfully treated with defibrillation.

On the ECG, signs of anterolateral myocardial ischaemia were observed, and the patient became severely symptomatic. The dissection, leading to total occlusion of the coronary lumen without distal antegrade flow, was classified as class F according to the National Heart, Lung, Blood Institute classification (NHLBI). A continuous infusion of nitroglycerin and heparin, as well as a lignocaine infusion, were administered. Within a few minutes, the patient’s condition stabilised and she was transferred to the cardiac surgery department.

On admission there, her systolic blood pressure was 110 mmHg and the heart rate was 80 beats/min. The patient complained of chest pains. Her cardiac necrosis markers were elevated: creatine kinase-MB (CK-MB) 99 U/l and troponin T (_Ths_) 449.4 ng/l. The continuous nitroglycerin and heparin infusion was maintained.

Transthoracic echocardiography (TTE) revealed hypokinesis of the apex and para-apical segments of the anterior, lateral and postero-inferior cardiac walls. The ascending aorta was 3.1 cm, the aortic bulb was 3.2 cm, and signs of dissection on the mitral side were observed. The ejection fraction was 52%.

**Fig. 2.**
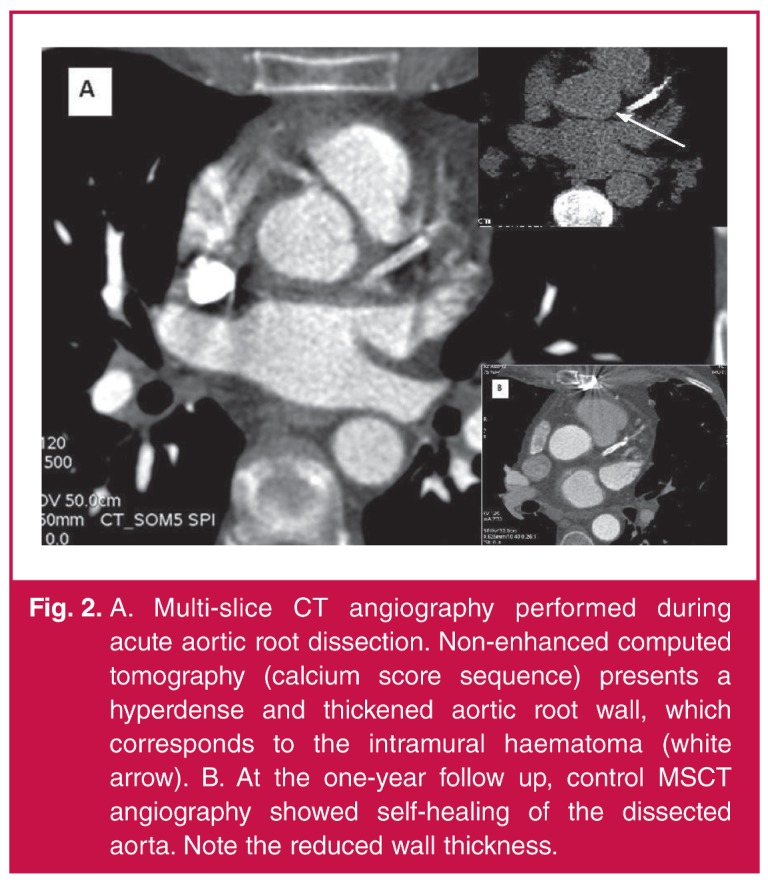
A. Multi-slice CT angiography performed during acute aortic root dissection. Non-enhanced computed tomography (calcium score sequence) presents a hyperdense and thickened aortic root wall, which corresponds to the intramural haematoma (white arrow). B. At the one-year follow up, control MSCT angiography showed self-healing of the dissected aorta. Note the reduced wall thickness.

Multi-slice CT (MSCT) angiography showed significant ostial LMS stenosis (intraluminal diameter 2 mm, area 5 mm^2^) without signs of atherosclerosis. Persistent staining of the left sinus of Valsalva, extending to the aortic annulus and non-coronary sinus of Valsalva, was found. The scan indicated limited aortic dissection (Fig. 2A).

The 12-lead ECG indicated anterolateral STEMI. Laboratory tests showed significant increase in levels of cardiac markers: CK-MB 538 U/l and T_hs_ 7 034 ng/l. A decision was made to intervene surgically. The time period between the iatrogenic dissection and surgical intervention was approximately six hours.

An intra-operative view confirmed the presence of a dissection of the left and non-coronary sinuses of Valsalva. There was also a haematoma along the proximal portion of the LAD, which extended to the surrounding epicardium. On a beating heart, without extracorporeal circulatory assist, total arterial myocardial revascularisation with double skeletonised internal thoracic arteries was performed. The right internal thoracic artery (RITA) was anastomosed to the LAD. The left internal thoracic artery (LITA) was anastomosed to the Cx. Further hospitalisation was uneventful.

At the one-year follow up, the patient was feeling well and remained asymptomatic. Control MSCT angiography (Fig. 2B) revealed complete healing of the limited aortic and LMS dissection. Competitive native blood flow, the LITA graft occlusion and the patent RITA graft were also seen on MSCT angiography.

## Discussion

Iatrogenic dissection of a coronary artery during a percutaneous procedure can be triggered by many factors, including unusual anatomy of the LMS, atherosclerosis of the LMS, difficulty when introducing a catheter, vigorous contrast infusion, inexperience of the operator, catheter type, inappropriate catheter position or sub-intimal passage of the guidewire.^5^

The choice of treatment strategy in the case of an iatrogenic coronary artery dissection depends on many factors, including haemodynamic stability, the patient’s clinical state, extension of the dissection, the number of dissected vessels, and SV involvement.^5^ When dealing with an LMS dissection, urgent surgical myocardial revascularisation is preferred. Many authors emphasise the unpredictable nature of a dissected flap.^6^

Although stenting gives the opportunity of a fast and direct approach, more than one stent is usually required to obtain proper blood flow in the LAD and Cx. Furthermore, percutaneous angioplasty may be associated with extension of the dissection area. While forming a haematoma, the coronary artery lumen may become occluded. In a long-term follow up by Unal and co-workers, after salvage PCI of the dissected LMS, rates of in-stent restenosis and repeated revascularisation were high.^6^ In addition, LMS dissection can easily affect the main branches, causing ischaemia in a large mass of myocardium, and clinical recurrence of angina pectoris.^7^

In our case, it seems that the previously implanted BMS provided blood flow through the LAD but the dissection resulted in haematoma formation. Although an attempt at covering the dissection with a stent was undertaken, recurrent VF occurred with each introduction of the catheter. There was no opportunity to protect the dissection percutaneously, therefore, a clinical approach focused on the patient’s stabilisation and transfer to the cardiac surgery department.

The results of laboratory tests and changes in the ECG suggested progression of ischaemia. In addition to recurrent VF, it showed that there was only temporary haemodynamic stability. In such a scenario, surgical treatment, especially in the case of limited aortic root dissection and persistent myocardial ischaemia, was the only reasonable and possible solution. ECG evolution indicated that the haematoma along the LAD could have been causing occlusion of the medial and distal segments, and anterior myocardial ischaemia.

Iatrogenic aortocoronary dissection (IACD) may be treated conservatively, especially in cases of high-risk patients, provided that entry of the dissected coronary artery is covered with a stent and the patient can be carefully monitored.^8^ On the other hand, IACD is unpredictable by nature. A stent implantation may not prevent type A ascending aortic dissection early after the primary procedure, therefore, sudden clinical deterioration may be observed.^9^ Spontaneous resolution of the SV dissection, even within 24 hours post procedure, has also been reported.^8^ Some authors suggest the surgical approach when the dissection extends into the ascending aorta for more than 4 cm.^1^

A decision on total, no-touch arterial revascularisation was made during the surgery. It allowed the blood supply to the ischaemic myocardial areas to be restored without manipulation of the ascending aorta. An unchanged sino-tubular junction and ascending aorta allowed SV repair to be avoided.

The one-year follow up and results of the control MSCT angiography confirmed the appropriateness of the intra-operative decision. Healing of the dissection probably caused competitive native and bypass flow, which contributed to occlusion of the LITA graft.

The percentage of self-healing dissections is unknown, as is the mechanism of this process. Almafragi *et al.*^10^ suggested that the healing of a coronary artery dissection is stimulated by retrograde flow and intravascular pressure augmentation caused by a bypass implantation. This corresponds with the high rate of in-stent restenosis. Our decision was confirmed by a favourable outcome in the patient, and the positive impact of the competitive flow.

## Conclusions

IACD poses therapeutic difficulties and individual risk evaluation may confirm the treatment strategy. Surgical intervention limited to myocardial revascularisation, performed as a no-touch technique, and conservative management of the limited aortic dissection may give satisfactory long-term results. Careful patient follow up is also required.
